# Teaching Microbiome Analysis: From Design to Computation Through Inquiry

**DOI:** 10.3389/fmicb.2020.528051

**Published:** 2020-10-29

**Authors:** Gail L. Rosen, Penny Hammrich

**Affiliations:** ^1^Ecological and Evolutionary Signal-processing and Informatics (EESI) Laboratory, Electrical and Computer Engineering, Drexel University, Philadelphia, PA, United States; ^2^School of Education, Drexel University, Philadelphia, PA, United States

**Keywords:** bioinformatics, microbiome, metagenomics, microbial ecology, multidisciplinary education

## Abstract

In this article, we present our three-class course sequence to educate students about microbiome analysis and metagenomics through experiential learning by taking them from inquiry to analysis of the microbiome: Molecular Ecology Lab, Bioinformatics, and Computational Microbiome Analysis. Students developed hypotheses, designed lab experiments, sequenced the DNA from microbiomes, learned basic python/R scripting, became proficient in at least one microbiome analysis software, and were able to analyze data generated from the microbiome experiments. While over 150 students (graduate and undergraduate) were impacted by the development of the series of courses, our assessment was only on undergraduate learning, where 45 students enrolled in at least one of the three courses and 4 students took all three. Students gained skills in bioinformatics through the courses, and several positive comments were received through surveys and private correspondence. Through a summative assessment, general trends show that students became more proficient in comparative genomic techniques and had positive attitudes toward their abilities to bridge biology and bioinformatics. While most students took individual or 2 of the courses, we show that pre- and post-surveys of these individual classes still showed progress toward learning objectives. It is expected that students trained will enter the workforce with skills needed to innovate in the biotechnology, health, and environmental industries. Students are trained to maximize impact and tackle real world problems in biology and medicine with their learned knowledge of data science and machine learning. The course materials for the new microbiome analysis course are available on Github: https://github.com/EESI/Comp_Metagenomics_resources.

## Introduction

In recent years, there has been a call for greater data literacy in life science education (Gibson and Mourad, 2018). Bioinformatics core competencies have been identified by various organizations. Competencies include a combination of biology, understanding of technologies, statistics, and computational methods in addition to teamwork, communication, and the scientific discovery process. Also, researchers have found that while learning the breadth of biology, computation, and math, it is important to start early and maintain depth and focus on a multidisciplinary topic (Anton Feenstra et al., 2018). Thus, it is concluded a series of courses, if not whole training program, is needed to effectively train students in bioinformatics. Also, an iterative teaching approach allows students to incorporate feedback, especially from multiple sources (e.g., biology and computation) (Marbach-Ad and Marr, 2018).

Metagenomics has been introduced in the undergraduate and graduate curriculums, but usually as a short course (Falana et al., 2015; Bolyen et al., 2019), research module in a larger course (Muth and McEntee, 2014; Gibbens et al., 2015; Lentz et al., 2017), or a single course (Edwards et al., 2013). Also, there is an issue of students from more biological disciplines and from more computational/engineering disciplines both gaining valuable knowledge from these courses.

To address some of these issues, we introduce three interdisciplinary courses to educate students in the realms of genomics, molecular evolution, and the bioinformatics analyses of genes and genomes. Students participating in these courses come from biology, biomedical engineering, electrical engineering, and computer science, providing a diverse multidisciplinary environment with great potential for peer learning. While developing hypotheses, students gain hands-on skills in DNA sample preparation and sequence analysis in the Molecular Ecology Laboratory and Bioinformatics courses. They analyze amplicon and metagenomic datasets that they helped to generate, using these to test hypotheses about microbial ecology, symbiosis, and the roles of microbes in nutrition and disease. Through the thematic activities, we actively engage students in the learning process, helping them to develop as critical-thinkers who understand the scientific method. The course sequence is complementary in its approaches, with the Molecular Ecology Lab being hypothesis generating and learning lab techniques, while the Bioinformatics course builds skills through a more traditional format, and the sequence finally culminates in the Computational Microbiome Analysis course where students share and learn about cutting-edge tools. Specifically, in the microbiome course, students conduct tutorials to learn cutting-edge tools by (1) independently following or composing tutorials, demonstrating what they learned, and sharing with the tutorial and results others, (2) learn from peers’ tutorials, and (3) learn the steps to analyze their project data. We attempt to reach out to heterogeneous backgrounds by having students take a hands-on lab course (rather than bio theory), by teaching bioinformatic algorithms through demonstration, by teaching coding through example and debugging, and through group work in two of the courses. We are the first to broaden training in microbiome data analysis so that students gain deeper understanding from learning bioinformatics basics to more advanced analysis via inquiry. Quantitative assessments of knowledge gain of 45 undergraduate students showed that students generally improved knowledge in several bioinformatics areas.

## The Structure of the 3-Course Sequence

Drexel university has 3 quarters (approximately 10 weeks each) per year. The course sequence is as follows: Molecular Ecology Lab and Bioinformatics are concurrently offered in the first quarter, followed by Computational Microbiome Analysis in a second quarter. Due to some life events, we offered the course sequence twice—once in the 2015–2016 and again in the 2016–2017 school years. In 2015–2016, the concurrent Molecular Ecology lab and Bioinformatics was offered in the Fall with the Computational Microbiome Analysis course in the Spring, while the second time, it was offered in the Fall/Winter. The specific learning objectives of each course are (1) Molecular Ecology: Proficiency in molecular lab techniques and knowledge of technologies, mastery of knowledge of computational analyses of ecology, and understand an application, methods, and synthesize hypotheses; (2) Bioinformatics: Be able to modify python code, introduced to bash scripting, learn algorithms such as dynamic programming, hidden Markov models, phylogenetics, and learn about their implementations (e.g., BLAST); and (3) Computational Microbiome Analysis: working knowledge of bioinformatics programming, proficiency in bioinformatics pipeline development, and learning how and when to use comparative genomics tools.

**FIGURE 1:**
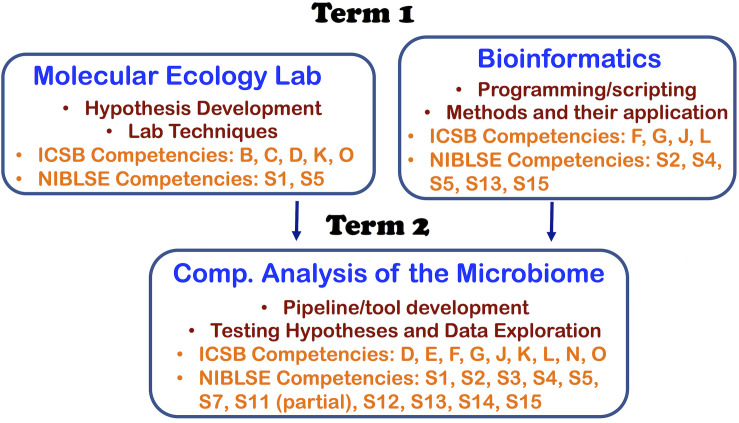
Each course in the sequence and its mapping to ICSB and NIBLSE competencies.

With the three courses, we were able to address 11 out of 16 core competencies identified by the Intl. Consortium for Systems Biology (ICSB) curriculum task force (Mulder et al., 2018) and 11 out of the 15 core competencies identified by Network for Integrating Bioinformatics into Life Sciences Education (NIBLSE) (Wilson Sayres et al., 2018). This course series teaches ICSB core competences—B: Depth in at least one area of biology, C: Biological data generation technologies, D: Details of the scientific discovery process and the role of bioinformatics in it, E: (at a high-level due to undergraduate curriculum): statistical research methods, F: bioinformatics tools and methods, G: ability of a computer-based system to meet scientific problem, J: Command line skills and scripting, K: Web-based Bioinformatics, L: Impacts of bioinformatics/genomics, N: (partial) communication of results to peers, and O: Effective Teamwork. We also address NIBLSE’s core competencies: S1: Role of Bioinformatics in hypothesis-drive biology, S2: Bioinformatic computational concepts, S3: Statistics, S4: Accessing genomics, S5: Using genomic tools, S11 (partial through functional prediction module): Using pathway prediction tools using expression tools, S12: Metagenomics, S13: Scripting, S14: Using software packages, and S15: operate different computing environments. A summary of the core competencies targeted in each course are shown in Figure 1.

### Molecular Ecology Lab

**FIGURE 2:**
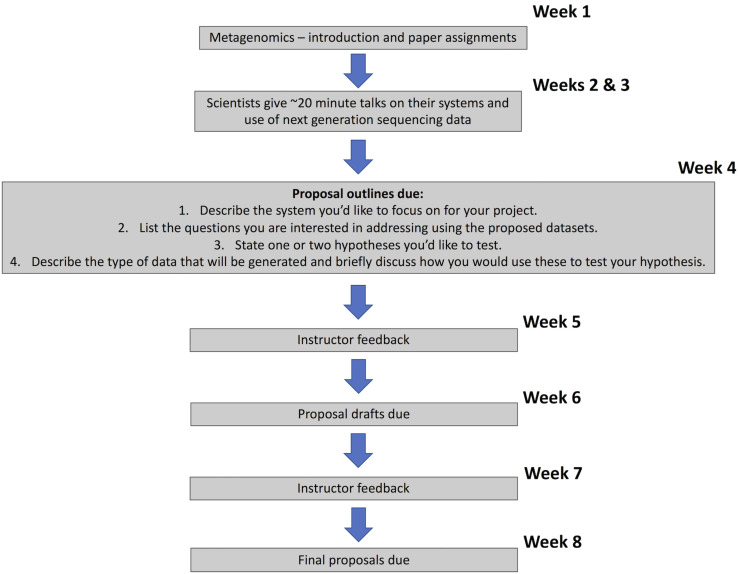
Timeline of the molecular ecology lab projects.

The Molecular Ecology Lab course (first quarter class in the sequence) was designed to train students in basic laboratory techniques and technologies from the field of molecular biology, applying these to enable research on microbial symbionts of animals. The course was also designed to emphasize the design of hypotheses and experiments using amplicon and meta-genomic/transcriptomic sequencing to ask questions about host-microbe interactions that are challenging to study in other ways. The timeline for the course project instructions is shown in Figure 2.

In this course, students were graded on: (1) two quizzes, which emphasized their understanding of methods/technologies and situations in which to apply them; (2) course participation, which included a requirement that the students demonstrate competency in DNA extraction, PCR amplification, PCR primer design, and gel electrophoresis; (3) an 8 page paper in which they analyzed and reported data that they generated on a bacterial endosymbiont of ants, showing competency in DNA sequence alignments, BLAST searches, and phylogenetics; and (4) their 4–6 page microbiome analysis proposal. Skills emphasized in the class were, thus, not only related to lab techniques but also thinking like a scientist and analyzing and interpreting data.

#### Molecular Ecology Project Proposals

For the microbiome analysis proposal students submitted one outline and one rough draft, using instructor feedback to improve their ideas, hypotheses, justification, and methodologies. We focused on five research programs that were put forth as areas where the students could develop questions that they could then test through a follow-up course: (1) reciprocal impacts between non-alcoholic fatty liver disease and gut bacteria; (2) identifying function of ancient gut symbionts of predatory army ants; (3) studies of ant gut microbiome gene expression in response to dietary variation; (4) microbial source tracking in the Delaware River watershed; and (5) studies on bacteria co-colonizing bioreactors with algae.

Scientists from labs supporting these projects delivered 20–30 min presentations at the start of the course, helping to establish the “menu.” They put forth knowns and unknowns for their systems, helping to make clear the motivations for study. For each presentation one or more articles from the primary research literature were assigned for background reading, helping students to develop further understanding of these subdisciplines.

Students were given some guidance in narrowing down the list of potential projects. As an example, see the below excerpt from the microbiome analysis proposal guidelines provided to the students:

“The best hypotheses will combine a mixture of novelty and realism, with clear links to mechanism as a guiding force or focus. For instance:

1. For the *Cephalotes* transcriptome project (project 3), one might hypothesize particular genes and pathways that should show transcriptional responses to the various diets if bacteria do indeed use substances contained within. One might also hypothesize which organisms to be involved.
2. For the army ant project (project 2), one might hypothesize functions expected to be common among gut symbionts of carnivorous animals. One might also propose functions that should differ between closely related strains of bacteria hosted by sibling ants belonging to the same colonies”.

While biologically-inquisitive students went through several rounds of hypothesis development with the instructor, those who were less-developed to choose hypotheses were given a specific problem with limited choices on hypotheses. Groups were encouraged to be heterogeneous, meaning that groups that contained at least bioscience and one engineering/comp student were encouraged for peer learning. All groups were required to submit a 4–6 page proposal draft that utilized metagenomics, metatranscriptomics, or 16S rRNA amplicon sequencing to study one of the potential projects presented in class. Students learned about the subject area through independent study and interaction with the instructors to learn more about these systems and techniques.

Examples of Specific Aims and hypotheses from undergraduate projects included:

Project 1

“Hypothesis 1: Non-alcoholic fatty liver disease development will correlate with changes associated with increased short chain fatty acid production.”

“Hypothesis 2: Non-alcoholic steatohepatitis progression may correlate with endogenous alcohol production.”

*Projects 2 and 3* – One student combined two of the projects on the menus.

“I predict that different amounts of Enzyme Commission numbers (E.C.s) associated with in (*sic*) digestion will be present in ants with different feeding types, as was found in Muegge et al. (2011)…. enzymes used in amino acid synthesis will be more common in *Cephalotes* than army ants because of the nitrogen poor diets in *Cephalotes*” (*Student is using precedent from a prior publication and knowledge of ant biology to predict differences in the devotion of gut microbes to particular digestive processes.*)

Project 3

“The main aim for this project is to find whether particular genes are highly expressed based on the diet. In this project, we’ll analyze metabolic pathways that should show transcriptional responses to various diets.”

Project 4

“The primary objective of the study is to identify microbes present in the watershed that correspond to specific sources of fecal contamination for MST. To achieve this, fecal samples have been gathered from a variety of microbial hosts at different times of the year, and water samples have been collected upstream and downstream of the potential contamination sites.”

### Bioinformatics

While students engaged in the Molecular Ecology lab, students took Bioinformatics, which was co-taught by Dr. Rosen (Engineering) and Dr. Russell (Biology). Most of this course was developed prior to the grant, except for the first 2-week coding bootcamp. Previously, the course had lacked some of the more practical data wrangling and retrieval necessary to start in bioinformatics. So, for the grant, we introduced an intensive introduction to bash and Biopython (Cock et al., 2009). The first 2 weeks were a review of molecular evolution, and a “coding bootcamp” that was an introduction to Biopython and the bash environment/job queuing system on Proteus, Drexel’s campus computing cluster (over 2000 CPU-cores offered to the campus community in 2014) (URCF, 2019). One of the programming assignments was to debug Biopython code to NCBI retrieve sequences, where intentional errors were introduced into the code that students had to correct. This exercise was specifically designed for the course and reinforced the idea that most bioinformatics programming is not coded from scratch, but that “related code” can often be found online (e.g., on a forum) and that it must be manipulated for specific solution to solve a specific problem. Subsequently to the coding bootcamp format, the biological goals and algorithmic foundations of dynamic programming/BLAST, hidden Markov models, phylogenetics, and sequence logos to represent DNA variation, were taught. Our lectures were structured so that the biological application and goals were laid out, followed by the computational and mathematical underpinnings of the algorithms. The course contains 3 homeworks, one midterm, and one final.

### Computational Microbiome Analysis

Computational Microbiome Analysis (also listed as “Statistical Analysis of Genomics” to enroll a wider audience) is the flagship course developed for the project. The course generally teaches fundamentals in the first 3–4 weeks; first, there is a review of shell scripting, Biopython, and running code in a cluster queuing environment (overlap with Bioinformatics for students that repeat). Then, an introduction to the microbiome (including the significance of the 16S rRNA gene), microbial ecology, and metagenomics is introduced. Large-scale databases and meta-analysis programs for both amplicon sequencing and metagenomics datasets [like QIIME (Bolyen et al., 2019) and MEGAN (Bağcı et al., 2019)] are covered. These fundamentals are expected to get students comfortable with automating code and using third party software, with both being necessary for the individualized course projects. Students also sign up for one or two tutorials, in which they must learn a particular package/method in-depth and present a summary of how the method works and give an example of how to run the software and the output that one can expect. While undergraduates present on 1 tutorial and graduate students present on 2 tutorials in groups of 2–3, most of the quarter (6–7 weeks) is consumed by the 10–12 tutorials from groupings of all the students. Usually, the instructor gives a 30 min lecture to give background on the analysis theme for the week, such as “Metagenome assembly,” which would explain the need and challenges of the area. Then, the rest of the week is 2 tutorials (usually 30 min in length on average) to talk about the algorithms and show how the various methods work, with added time for discussions. For our example theme week, this would include a review of IDBA-UD and Metaspades (depending on the year). The students work on instructor-selected datasets to demonstrate the tools in their tutorials and compare metrics, such as N50/min and max contig lengths for our example theme week. The students use online materials about the associated tools to develop the 10–15 min algorithm discussion followed by a 15–20 min tutorial demonstration. While a few groups do take the class through a real-time tutorial, usually 15–20 min is not enough, and the students, who are teaching, usually point the students, who are learning, to a Github repository where they can view and run the code themselves. This course focus on tutorials of important microbiome analysis tools allows the course to update itself and keep up with the quickly-moving field of microbial community analysis. Tutorials have included High-throughput Phylogenetics [using alignment and tree methods on CIPRES (Miller et al., 2012), learning microbial ecology comparison techniques (like diversity metrics, distance measures between samples like Unifrac (Lozupone and Knight, 2005) etc., ordination, etc.], assembly and binning of genomes from metagenomics, taxonomic identification from metagenomics, functional annotation of metagenomes, functional prediction of amplicon data, metatranscriptomic analysis (differential abundance comparisons), and even basic statistics (like ANOVA/MANOVA/correction for multiple comparisons) and analysis like gene set enrichment analysis. The tools that are reviewed can change from course iteration to course iteration. For example, tutorials on taxonomic classification methods went from Metaphlan2 (Segata et al., 2012) in the first year to Kraken2 (Wood et al., 2019) and Kaiju (Menzel et al., 2016) in the latest iteration.

The course projects are the most important aspect of this course. Students who take the Molecular Ecology lab will analyze a dataset that they set out to investigate to verify a hypothesis. Students, who did not take the Molecular Ecology lab, can choose from a menu of datasets and project ideas, some of which may be investigating algorithms and comparing methods (which appeal to the engineering and computer science students in the course.) Students received detailed guidance from the PIs and teaching assistants (TAs). Also, we made a concerted effort to pair graduate students with undergraduates, so that each team had a balance of levels. Projects titles include (results and project findings can be found on the course Github page):

1. “*Metatranscriptomic Analysis of Laboratory-reared Cephalotes varians RNA Dataset and Comparison across Four Dietary Treatments*.
2. *“Metagenomic analysis of and comparison between the photosynthetic microbial communities in two photobioreactors”*.
3. *“A Metagenomic Analysis of Healthy Mice vs. Fatty Liver Disease Induced Mice on Both Control and High Fat Diets”*.
4. *“Finding Patterns in Time-course Metagenomic Data”*.
5. *“Metagenomic Analysis of Army Ant Guts”*.
6. *“Building Ensembles of Taxonomic Classifiers”*.

Each week, students had to compose quiz questions (with corresponding answers), which we found acted as a formative assessment, to understand what students were absorbing from the lectures and tutorials since this forced students reflect on the material in weekly intervals. Undergraduate students learn one tool in-depth by teaching a tutorial, and finally, most of the skills are learned from a data analysis project. In order to keep this projects on-track, we have learned that students need to submit a project declaration, proposal, progress report, and final report throughout the short 10-week quarter.

## Project Outcomes

A total of ∼150 students enrolled in all three courses for the two offerings. However, we performed formative and summative instruments (a demographic questionnaire, de-identified but non-blind comparison of pre- and post-surveys; and bi-weekly administered surveys) only for the undergraduates. The surveys were administered under instruments approved under Drexel IRB #1211001675, and we obtained student consent at the beginning of each course. *Forty*-five undergraduates enrolled in at least one of the three courses, with 4 taking all three (there were substantially more graduate students that took all 3 courses). We surveyed demographics of the 45 undergraduates that took at least one of the courses, with 62% of them identified as male and 6% identifying with an ethnic group that was not Caucasian or Asian.

From a pre-course survey, students were asked to rate their abilities/skills of different subjects. In Table 1, Most students rated themselves with no skills in metagenomics, bioinformatics, genetics, and hypothesis development. This has identified that focusing the course on such skills is much needed.

**TABLE 1:** Student self-reported knowledge and skills (*n* = 45).

Level of skill	No skill	Somewhat skilled	Very skilled
Genetics	31%	49%	20%
Ecology	51%	38%	11%
Bioinformatics	51%	42%	7%
Metagenomics	80%	13%	7%
Hypothesis development	31%	47%	22%
Experimental design	16%	51%	33%
Programming	18%	53%	29%

### Reflections From the Molecular Ecology Lab

From the Molecular Ecology Lab course, we generated four new next-generation sequencing datasets. These were presented to students in the Computational Microbiome Analysis follow-up course, a class whose roster included several students who participated in the lab.

Beyond serving as a prelude to the Computational Microbiome Analysis course, and an introduction to how the ‘omics revolution has revolutionized microbiology, the microbiome analysis proposal served to allow students to “demonstrate a capacity to synthesize and integrate results into the broader context of the field,” an objective from the course syllabus (all syllabi can be seen in the Supplementary Material). Through in class discussions, rough draft feedback, it was clear that students were able to do this to some extent. While some strongly mimicked documents disseminated from the scientists leading these projects, others demonstrated a strong vocabulary and independent thinking in areas they had not previously studied.

Through assessments of student quizzes and papers, it was clear that all developed a deeper understanding of microbial ecology and the applications of DNA/RNA sequencing to study microbes in their natural habitats. Several showed clear proficiency in developing well-justified hypotheses and aims. At minimum, all were able to develop a coherent and reasonable set of research activities.

Challenges included the fact that students often deviated from directives to limit their proposed work to suit the available/pending datasets. This meant that for those moving on to the subsequent Computational Microbiome Analysis course, several could not directly test their hypotheses.

Another challenge was the very steep learning curve required for students to develop a good understanding of bacterial metabolism. This was key to formulating strong hypotheses for several of the projects and more time devoted to this area during the course would have been immensely helpful.

### Reflections From Bioinformatics

The Bioinformatics class was the most standard class of the three, with homeworks and tests. The biology students found the coding challenging but rewarding, with the statement *“*…*coding activities most difficult to understand but most rewarding” and “*…*use of NCBI was great.”* Others wanted to see more coding and did not want the theory behind the algorithms – “*I expected to learn more practical skills that I can use such as a script to sequence alignments but this course taught a lot about background theory of these algorithms.*”

Many students were satisfied with the course – *“The fusion of disciplines is readily apparent”, “This course is more hybrid than all other engineering science courses I‘m taken. Requiring understanding of two fields to apply them in bioinformatics”.* There was a trend that students with backgrounds in biology found programming part challenging and the students with programming background found biology challenging.

### Reflections From Computational Microbiome Analysis

In the computational microbiome analysis course, students learned about state-of-the-art methods and tools used for microbiome and metagenomic analyses through hands-on tutorials and projects. Because each tool could possibly elicit a few weeks to itself alone, it is perceived that too much is covered in the class. We required that each student group spend half of a 30 min slot on describing how the method/tool works and half the time showing how to operate the tool and interpret its results. We did notice that computational students seemed to spend more time on the methods while biological students spent more time on results interpretation, which is to be expected. The hope is that the tutorial will give a basic introduction to the students, so that they can be aware of its existence in the vast toolbox of microbiome analysis to reference and learn more in-depth when needed.

The tutorials, each learned in-depth by a few students, were reinforced to the rest of the class through reflection – students were required to hand in 3 mock quiz questions and answers, some of which would be selected (or reshaped into more cohesive questions) for a quiz given the following week. The weekly quizzes were a good mechanism, as it induced a “studying for the quiz” reinforcement of the material. In our second iteration of the three-course sequence, we limited quiz content to conceptual understanding of the tool’s purpose and interpretation of their function. This way, students could focus their studying and understand the fundamental concepts of each week’s theme.

While students are excited by no tests or finals, they soon realize the curse of a project-based course, as it is 50% of their grade. As with all projects, students struggle to maintain a schedule, so we have found that 10-week project-based classes need multiple hard deadlines throughout the course to keep students on track. Having four deadlines is perfect. The “Project declaration” (due in week 2) is where the students must decide which topic they are interested in and demonstrate that they can gather the data. Demonstrating that students can import data structures and objects is pivotal, as we have found that many groups delay actually working with the data. Then the “Project proposal” (due in the week 5) must (1) describe the problem they are interested in (they would be able to take this hypothesis development directly from the Molecular Ecology Lab if enrolled in this class prior or if not, detail their hypothesis or design idea) and (2) propose the analysis steps and timeline of how they will test their hypothesis or build a tool. Then, the “Progress Report” (week 7) gives a deadline that students must report on some analysis steps, any issues encountered, and gives them the final chance to modify their proposed analysis design. Around week 10, students must give an oral presentation on their final results, and the following week, a written report is due. These spaced deadlines keep students thinking and working on the project in a timely manner.

Many undergraduates find that the freedom from tests and finals is more challenging than they expect, because they must now “get things to work” and peruse literature to understand concepts and tools. Varying quality of the tutorials and projects result. However, instead of teaching and testing on methods that are in constant flux, the focus is software pipeline design to test hypotheses or make tools, which builds critical thinking. Some students realize that this course helps build skills needed in the workforce. A spontaneous email that was received approximately 6 months after the Computational Microbiome Analysis course by a graduate student, who went on to work in the pharmaceutical industry, wrote:

*“Dr. Rosen*,
I would like to thank you in the strongest possible terms for your course in the Spring term of ’15: ECES 690.
Without a doubt it is the single most applicable course I have taken, not only at Drexel, but in my entire academic career, to my current endeavors.
At the time I expected it to be useful, but now I am discovering that the lessons learned there are ^∗^completely indispensable^∗^ to my occupation.
I encourage you to keep up the amazing work with that class, and more like it, so that a new class of students can benefit from such instruction as I have had”.

### Assessment of Learning Outcomes

We can show that bioinformatic competencies generally improved upon completion of any of courses in the three-course sequence. Pre- and Post- surveys of the Bioinformatics and Computational Microbiome Analysis classes included 20 content questions; the full list of questions can be found in the Supplementary Material. Quantitative data was collected by using a pre- and post-survey that was administered at the beginning and the end of the course and were coded so only the evaluator knew the identities. The questionnaire consisted of 20 open-ended content questions (seen in questions.docx in the Supplementary Material) on the microbiome, metagenomics and molecular ecology. The student responses in both the pre- and post- surveys were graded independently by two subject matter experts on a scale 1–5, with 1 meaning that the student demonstrated no knowledge and 5 meaning that the student demonstrated excellent mastery of the material. The pre- and post- surveys were collected from the 45 undergraduate students who agreed to participate in the study with 12 pre- and post- matched surveys that were near-completely filled out (due to student absences or incomplete surveys on either end since the surveys were lengthy). There were 7 questions that received more than 10 responses on the pre- and post- surveys and were statistically significant (as determined by a 2-tailed *T*-test). Other questions either received less than or equal to 10 responses or they were not significant (meaning that there was no statistical difference between the pre- or post-survey answers). The content questions that were statistically significant are:

2. What is a Standard Flowgram File and what type of DNA sequencer outputs it?
3. How would you convert a SFF file to a FASTA file?
4. What is the difference between PCA (Principal components analysis) and PCoA (Principal coordinates analysis)?
5. What are the trade-offs of supervised learning algorithms (trade-off of random forests vs. support vector machines vs. bayes classifiers)?
9. Genome sizes for a given species or taxon vary, often considerably. Describe why metatranscriptomic reads need to be normalized, especially for downstream analysis.
14. Name at least two ways that you can annotate WGS (whole-genome shot sequencing) reads with functional annotation?
15. Describe the difference between phylogenetic tree reconstruction methods?

**FIGURE 3:**
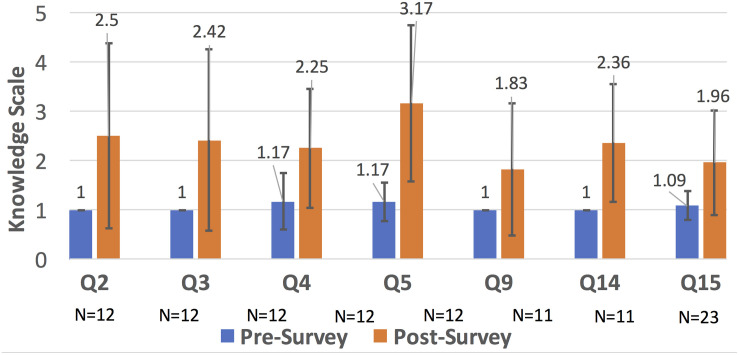
Bar chart comparison of the knowledge scale for different bioinformatic topics (that were statistically significant). In around 7 areas (many related to microbiome analysis), there was improved knowledge. Other areas, see Supplementary Material, were not noticeably improved due to removal because of curricular changes, lack of enough responses, or no significance between the pre- and post- surveys.

As seen in Figure 3, Question 5 (about machine learning algorithms learned in Comp. Microbiome Analysis) has the biggest increase in understanding. Questions 2, 3, and 15 were learned in Bioinformatics, and Questions 4, 5, 9, 14, and 15 were learned in Computational Microbiome Analysis (note that question 15 was taught in both classes). Students completed the lab assignments, proposal report, computational assignments, tutorial demonstrations, and project demonstrations that meet the criteria in Figure 1. Students gained knowledge of wet lab and programming techniques, although proficiency was lacking for students from the opposite discipline, and this was a challenge. However, most students gained an appreciation for algorithms through hands-on calculations and learning how to use a tool through tutorials. Finally, microbiome analysis skills through group projects were facilitated through peer learning, and students gained at least some skills/knowledge that they did not have before. This demonstrates that knowledge of bioinformatics and metagenomics analysis increased for some topics. We believe that knowledge increased for other questions, but the sample size was too small (due to content question changes and not as many students answered those questions).

We have also included a qualitative report on student perceptions, experiences, and understandings (seen in the Evaluator_report.pdf in the Supplementary Material) that can elucidate more detail on how the learning outcomes were realized by the students.

## Discussion

We describe a 3-course sequence in microbiome analysis training via a Molecular Ecology Lab, Bioinformatics, and Computational Microbiome Analysis. A summative analysis and student feedback demonstrate that the course sequence and individual courses had some beneficial impact on student bioinformatic competencies. In a world where data is becoming ever abundant, students need to be equipped with the knowledge to handle it. Our training sequence helps to meet those training goals. Yet, there is still the challenge of educating students from heterogeneous backgrounds (biology and computation/engineering), so that students can (1) come to a level playing field or (2) speak each other’s languages to work together and learn from each other. Future work may involve iterative differentiated coursework, adding more peer learning to the bioinformatics class, offering short courses (or bootcamps) to facilitate interdisciplinary communication for peer learning (computational students to get up to speed on biology and biology students to improve their programming).

Training in an emerging multidisciplinary field, that has great potential, importance, and need, has both its advantages and challenges. We have found that students who have bioinformatic skills and understand the domain science are urgently needed in the workforce. We encourage faculty and administration at universities to look past immediate barriers (such as financial constraints and/or politics) and foster interdisciplinary teaching and courses. When successful, we can train a new generation of scientists and engineers who will push the boundaries of discovery.

## Data Availability Statement

The datasets generated for this study are available on request to the corresponding author. The Computational Microbiome Analysis course materials developed plus student projects and tutorials can be found at: https://github.com/EESI/Comp_Metagenomics_resources.

## Ethics Statement

The studies involving human participants were reviewed and approved by the Drexel University Institutional Review Board under project #1211001675.

## Author Contributions

GR formulated the concept of the three-course sequence, designed and offered the courses, and wrote most of the manuscript. PH conducted and summarized the summative student findings. Both authors contributed to the article and approved the submitted version.

## Conflict of Interest

The authors declare that the research was conducted in the absence of any commercial or financial relationships that could be construed as a potential conflict of interest.

## Supplementary Material

Supplementary Syllabus 1 Molecular ecology lab syllabus.

Supplementary Syllabus 2 Bioinformatics syllabus.

Supplementary Syllabus 3 Computational microbiome analysis syllabus (sometimes called Statistical Analysis of Genomics).

Supplementary Questions The questions that were a part of the IRB process at the beginning of the project. Some questions that assessed outdated topics were omitted. Also, questions that did not have a statistically significant difference between groups were omitted.

Supplementary Evaluator Report A qualitative report of formative assessments of the classes.
