# Supplementary material for: Teaching Microbiome Analysis: From Design to Computation Through Inquiry
Source: Front Microbiol. 2020 Oct 29;11:528051. doi: 10.3389/fmicb.2020.528051 (PMC7658192; doi:10.3389/fmicb.2020.528051)
Supplement: Supplementary Syllabus 1 — Molecular ecology lab syllabus. [file Data_Sheet_1.docx]

**Molecular Ecology Lab**

**Fall 2016**

**ENVS 327/527**

**2 credits**

**Time:** Friday 1:00 p.m. – 4:50 p.m.

**Location:** PISB 202

**Instructor:** Dr. Jacob A. Russell

**Phone:** 215-895-1643

**e-mail**: [Jacob.A.Russell@drexel.edu](mailto:Jacob.A.Russell@drexel.edu)

**Office:** PISB 325

**Office hours:** Thursday 11:00 a.m. – 1:00 p.m.

**TA:** Katie D’Amelio

**e-mail:** [cld64@drexel.edu](mailto:cld64@drexel.edu)

**Office:** PISB 121

**Office hours:** TBD

**Course Description:**

Genomes are known to vary between individuals, populations, and species. But until recently, the degrees and implications of this variation have not been appreciated. Fortunately, with the advent of fast, easy, and affordable techniques to isolate and study molecular markers, researchers have been able to quantify and harness this genetic variation, thereby addressing many previously intractable questions. This has resulted in an explosion of publications shedding insight into topics ranging from evolutionary histories and phylogenetics, to organismal diversity, speciation, behavior, conservation, disease, and forensics.

As such, within the discipline of organismal biology it has become important to both understand the logic and philosophy behind these molecular techniques and to develop a hands-on working knowledge of the methodology and statistics. In this course we will address the latter needs.

1) Through weekly lab exercises, students will develop fundamental hands-on skills in the techniques used in molecular ecology research. 2) Through development of a proposal designed to study microbial communities through metagenomics, students will also strengthen skills in hypothesis development, manuscript and grant writing, and experimental design. 3) Through computational exercises, students will learn how molecular data are analyzed.

**Course Readings and Resources**: journal articles TBA

**Course Purpose within the Program of Study:**

Molecular Ecology Lab can serve as a laboratory elective for students obtaining degrees in Biology or Environmental Science. The course conceptually fits well within the Ecology/Evolution/and Genomics concentration in the Biology major, although it also emphasizes organismal and molecular biology. The topics covered in the lab provide a follow-up to several covered in BIO 217, placing more of an emphasis on studies of evolution made from the molecular level. The course also follows up on topics from ENVS 230, 284, and 286, illustrating how the application of molecular tools can be used to understand major topics covered in these courses. The focus on microbial ecology also provides a complement to coursework on microbiology (e.g. BIO 221).

**Statement of Expected Learning:**

This course has three primary objectives:

1. To develop proficiency in several molecular laboratory techniques, including DNA extraction, PCR, and gel electrophoresis. Every student must demonstrate their abilities to successfully master these techniques during the quarter, based on the assessment of the TA and professor.
2. To demonstrate basic mastery of the computational analyses used in the study of molecular ecology. This will include an introduction to the NCBI website; quality control assessment, assembly, alignment, and analysis of DNA sequences; the construction of phylogenies; etc. Students will demonstrate their proficiency though their successful completion of computational analyses and data presentation (i.e. figures & tables) to be submitted at the end of the quarter with a short journal-style research paper.
3. To demonstrate a capacity to synthesize and integrate results into the broader context of the field. Achievement of this objective will be achieved by submission of a metagenomics research proposal that incorporates biologically informed hypotheses, while spelling out a clear rationale for the proposed research. This will also be assessed through a short journal-style research article focused on the results of the quarter’s research.

**Students with Disabilities:**

Reasonable accommodations are available for students with a documented disability. If you have a disability and may need accommodations to fully participate in this class, please talk to the professor **during the first two weeks.** Furthermore, to make the necessary accommodations, you will need to register with the Office of Disability Services (215-895-1401): <http://www.drexel.edu/ods/>

**ADD, DROP AND WITHDRAWAL POLICIES**

- You can **add** or **drop** this course until the end of week 1 (Sunday September 25 at 11:59 p.m.). For more on university policies relating to adding or dropping a course, see [http://drexel.edu/provost/policies/course-add-drop/](http://drexel.edu/provost/policies/course-add-drop/%20) .
  If you add this course after the start of the term, you are responsible for completing ALL work that you may have missed.
- The course **withdrawal** deadline is the Friday of week 7 (Friday November 11). You will have received some graded work prior to this deadline. If you have any questions about your progress at any time of the term, please contact me. If you choose to Withdraw, a “W” will be recorded in your transcript. For more on university policies relating to adding or dropping a course, see http://drexel.edu/provost/policies/course-withdrawal/%20/ .

**Attendance Policy:**

After an absence, students must contact me via e-mail (within 2 days of the missed class) to inform me of the cause for your absence. If the reason is excusable (e.g. illness, emergency, required travel), then there will be no penalty. Otherwise, students will lose 5% from their final grade for each unexcused absence. *Due to the fast-paced nature and once-a-week-meeting format of the course, it is very important that you make every effort to attend each lab.*

**Policy on Missed Deadlines:**

All unexcused late assignments will receive a 10% deduction per day late.

**Policy on Academic Dishonesty:**

For Drexel’s policy on academic integrity, visit:

<http://drexel.edu/studentaffairs/community_standards/studentHandbook/general_information/code_of_conduct/>

Except where group/team activity is required, it is assumed that ALL work is solely that of the individual student whose name is associated with the work. ANY form of cheating (copying, plagiarizing, using another’s work, permitting another student to use your work, falsifying data, etc.) will not be tolerated and can result in immediate disciplinary action.

Students who violate these policies (e.g. through cheating or plagiarism) will receive an F for the course and will be sent before the Drexel Office of Judicial Affairs:

<http://www.drexel.edu/judicial/default.html>

**General Code of Conduct:**

Students are expected to refrain from disruptive activity during class. Cell phones must be turned to vibrate (read below). Text messaging and phone calls are not allowed. Use of laptop computers and electronic devices must be limited to note-taking or in-class computational exercises. Students must also refrain from talking out of turn and may be asked to leave the class should they fail to abide by these rules.

**Lab Paper & Project:**

Students will summarize the results of the lab project in a 6-8 page (double-spaced) paper submitted by December 2. More detail will follow later in the quarter. Papers will include a brief introduction, a description of materials and methods, results, discussion, and references. Students will also use tables and figures to display results. This year’s project will focus on basic characterization of bacterial communities from a group of related predatory ants.

**Lab Quizzes:**

Students’ intellectual progress will be monitored by two quizzes that test their understanding of proper lab technique, methods, and analyses, along with experimental design and rationale. These will be a combination of short answer, essay, and multiple choice questions.

**Lab Notebooks:**

In accordance with good research practice, all students must keep detailed, hand-written notebooks. Failure to do so will result in a deduction from the class participation grade.

**Course Participation:**

It is very important that students attend all labs and that they pay very careful attention to experimental protocol, sample purity, data analysis tutorials, and guidelines for lab safety. Active participation in discussions is also encouraged.

**Graduate Project:**

Graduate students will work with the professor to develop an extra-curricular project that they will complete during the latter half of this course. Projects may be individualized, or they may involve group activity. The professor must approve each project, and will work with students to develop a contract on the criteria for grading and satisfactory completion. It should be noted that we will likely not have the time or space for students to work on these projects in class. Should such space be needed, students are encouraged to talk to the professor. In addition, funding for these projects may not be available through the funds used to run the course. It is imperative, then, that students working on separate projects identify a clear means of funding to support their proposed

work if the expenses go beyond what the course can cover.

-Proposals are due October 21. They should be 3-6 pages long (double spaced, 11-12 point times new roman, 1” page margins). They should include a background and justification (providing a broader context for the work you are doing and making it clear why one would want to study the project you propose), specific aims (what are the goals of this project; which goals will you accomplish for this class specifically), methods section, timeline section, expected results section, a section on your strategy for summarizing your data, and a references cited section (cite at least 4 primary research articles). This proposal will count toward 20% of the project grade. Please discuss ideas with the professor before submission.

-Progress reports are due November 11. These should be 2-4 pages long (double spaced, 11-12 point times new roman, 1” page margins). They should clearly indicate what you have accomplished, what you have yet to do, and where you have run into problems. They should also propose whether the objectives/aims need to be altered given any problems or opportunities arising from this work. This report is worth 10% of the project grade.

-Final reports are due December 9. These should be 6-8 pages long, not including any figures, tables, or references (double spaced, 11-12 point times new roman, 1” page margins*). You may reuse parts of the proposal for this final report* (e.g. background & justification, aims, methods). Most of the grade for this report will rest on the clear description of your actual methodology, clear indication of what worked vs. what went wrong and how you attempted to troubleshoot, the presentation of all data in a clear fashion (discuss requirements for your specific proposal with the instructor), a well-reasoned interpretation of the results, plus a future directions section on how you will use molecular tools to move the project forward. Sections to include are: background & justification, specific aims, methods, results, and future directions. Include a references cited section too, though this doesn’t count toward the proposal length (much like figures & tables). Again, cite at least 4 primary research articles.

**Metagenomics Proposal**

All students will write a 4-6 page proposal draft (double spaced, 1” margins, 12 pt Times New Roman font) that utilizes metagenomics or metatranscriptomics to study one of the systems presented in class OR your own system if deemed practical in consultation with the instructor.

A preliminary outline is due October 14.

The rough drafts are due October 28, 2014 at 12 p.m. (via TurnItIn).

The final drafts are due November 11.

Keep in mind that most of you will not carry out any of the proposed wet lab work (at least not as part of this class). Instead, others (e.g. our TA Katie D’Amelio) are already executing (or will soon execute) these experiments. Instead, your hypotheses will help to guide analyses that students perform in this Winter’s Statistical Metagenomics class, taught by Dr. Gail Rosen.

Here are 2 systems…we may announce more to come

-transcriptional responses of *Cephalotes* (turtle ant) gut bacteria to ant diets (metatranscriptomic dataset)

-metagenomes of New World army ants

This proposal should discuss the planned experimental design for your system (spelled out in files to be posted on the course website), the rationale for carrying out this work (which will include some background on the system; **read more papers!**), and the broader conceptual framework that the work addresses (**read more papers!**).

Specific hypotheses should then be put forth (look at the presentation pitches that we will make available online), but do some digging of your own to make predictions about gene functions or organisms that should vary in response to different “treatments”). The best hypotheses will combine a mixture of novelty and realism, with clear links to mechanism as a guiding force or focus. For instance:

-For the *Cephalotes* transcriptome project, one might hypothesize particular genes and pathways that should show transcriptional responses to the various diets if bacteria do indeed use substances contained within. One might also hypothesize which organisms to be involved.

-For the army ant project, one might hypothesize functions expected to be common among gut symbionts of carnivorous animals. One might also propose functions that should differ between closely related strains of bacteria hosted by sibling ants belonging to the same colonies.

Proposals should make it clear how hypotheses will be tested using sequencing and subsequent bioinformatics approaches, highlighting the expected outcomes that would support or argue against the hypothesis. They should also highlight the pitfalls of the proposed research, including the various meanings of negative results and how different explanations could be teased apart with proper analysis.

Sections and lengths are as follows:

-Background & Significance: **~ 1 page**

-Specific Aims (a section stating your main hypotheses and goals): **~1 page**

-Methods: **~1 page**

-Expected Results and Interpretation: **~1 page**

**Undergraduate Grading Policy (100 total points)**

*10 pts* course participation

*30 pts* quizzes

*30 pts* metagenomics project outline and proposal

*30 pts* lab paper on class project

**Graduate Grading Policy (100 total points)**

*10 pts* course participation

*30 pts* quizzes

*30* metagenomics project outline and proposal

*30 pts* lab paper on class project

*20 pts* graduate project (proposal, progress report, final report)

**Expected letter grade-breakdown:**

**A+** = 98% or more; **A** = 92 - 97.99%; **A-** = 90 - 91.99%

**B+** = 88-89.99%; **B** = 82 - 87.99%; **B-** = 80 - 81.99%

**C+** = 78-79.99%; **C** = 72 - 77.99%; **C-** = 70 - 71.99%

**D+** = 68-69.99%; **D** = 62 - 67.99%; **D-** = 60 - 62.99%

**F** = 59.99% or less

**
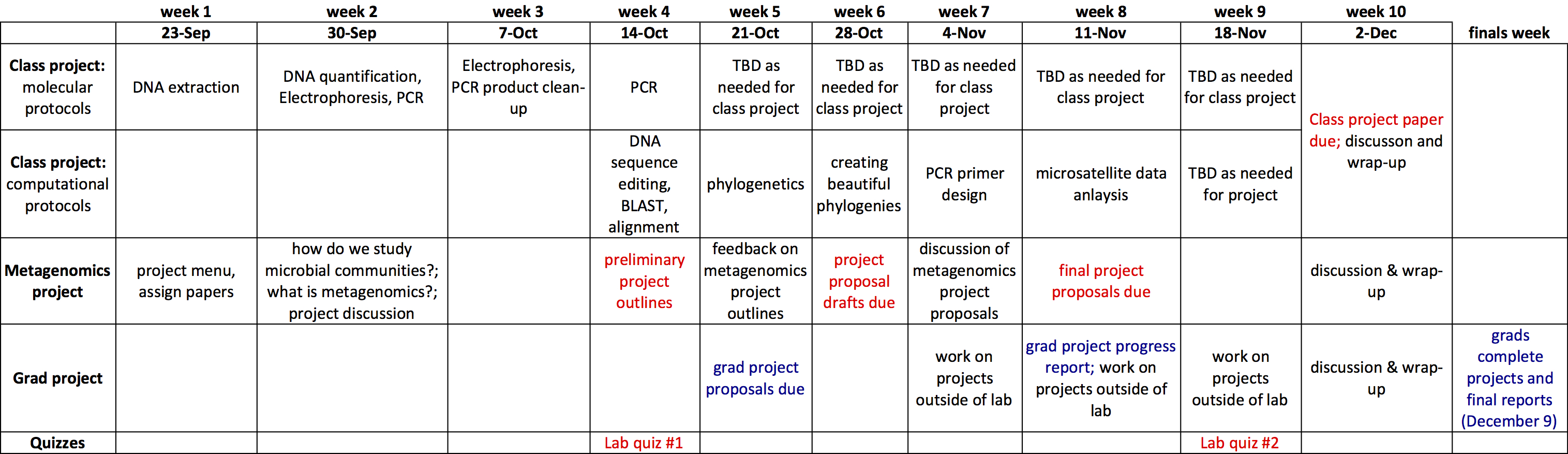
**
