# Supplementary material for: Teaching Microbiome Analysis: From Design to Computation Through Inquiry
Source: Front Microbiol. 2020 Oct 29;11:528051. doi: 10.3389/fmicb.2020.528051 (PMC7658192; doi:10.3389/fmicb.2020.528051)
Supplement: Supplementary Syllabus 2 — Bioinformatics syllabus. [file Data_Sheet_2.doc]

**Bioinformatics I**

**BIO 331 & 534/ECES 441 & ECES 640**

3 credits

Thursday 6:30 – 9:20 p.m.

PISB 215

**Instructors:**

Dr. Gail Rosen

gailr@ece.drexel.edu

215-895-0400

Bossone 403

Office hours: Tuesday 11-1

Dr. Jacob A. Russell

jar337@drexel.edu

215-895-1643

PISB 325

Office hours: Thursday 11-1

**Course Overview:**

This course will focus on developing the computational, algorithmic, and database navigational skills required to analyze genomic data that have become available with the development of high throughput genomic technologies. We will also illustrate statistical signal processing concepts such as dynamic programming, hidden markov models, information theoretic measures, and assessing statistical significance. The goals will be achieved through lecture and lab exercises that focus on genomic databases, genome annotation via hidden markov models, sequence alignment through dynamic programming, genome sequence assembly and annotation, phylogenetics, and comparative genomics.

**Statement of Expected Learning:**

1. To show competency in basic bioinformatics techniques related to genome assembly and annotation, DNA sequence alignment, and both comparative and evolutionary genomics. The achievement of this objective will be assessed through in class activities and homework assignments. For graduate students, this may also be exemplified in their project reports and presentations.
2. To show a working knowledge of the algorithms used for bioinformatics. To understand how algorithms can be innovated and how one can identify accurate parameters for use in these algorithms. Achievement of this objective will be assessed through in class activities, homework assignments, and problems on exams. For graduate students, this may also be exemplified in their project reports and presentations.
3. To demonstrate skills in database navigation, data extraction, and creation of custom databases for use in genome studies. This will be assessed through homework questions and problems on exams. For graduate students, this may also be exemplified in their project reports and presentations.
4. To demonstrate a thorough understanding of genomes, their features, how they vary, and the forces that drive this variation. This will be assessed through homework questions and problems on exams. For graduate students, this will be exemplified in their project reports and presentations.

**Pre-requisites**

While there are no formal pre-requisites, thought students must have taken at least introductory biology.

**Course Reading Materials**

We have no required textbook

**Expected letter grade-breakdown**

**A+** = 98% or more; **A** = 92 - 97.99%; **A-** = 90 - 91.99%

**B+** = 88-89.99%; **B** = 82 - 87.99%; **B-** = 80 - 81.99%

**C+** = 78-79.99%; **C** = 72 - 77.99%; **C-** = 70 - 71.99%

**D+** = 68-69.99%; **D** = 62 - 67.99%; **D-** = 60 - 62.99%

**F** = 59.99% or less

**Grading Policy:**

**Undergraduates (331/441)**

*15 pts* in-class activities

*45 pts* 3 homework assignments

*15 pts*  midterm exam

*25 pts* final exam

**Graduates (631/640)**

*15 pts* in-class activities

*45 pts* 3 homework assignments

*15 pts*  midterm exam

*25 pts* final exam

*10 pts*  graduate project written report

*10 pts*  graduate project oral presentation

**Details on specific assignments:**

***Graduate students from ECES 640 and BIO 631 -*** To earn your graduate credit you are required to conduct a small research project to be overseen by one of the instructors. After initial topic development and approval, students will submit project proposals by the start of week 3. Research topics may be focused on objectives relating to genomics (e.g. perform a comparative genomic study; identify conserved regulatory elements through phylogenetic footprinting), algorithm or software development, databasing, etc. Students will turn in a project update by the end of week 6. The final report is due the last day of class, when students will give 15 minute presentations on their projects. Please see “Grad Project Paper Guidelines.doc” on the course website for more details.

**General Code of Conduct:**

Students are expected to refrain from disruptive activity during class. Cell phones must be turned off or silenced (i.e. on vibrate). Text messaging and phone calls will are not allowed. Use of computers and electronic devices must be limited to note-taking or in-class computational exercises. Students must also refrain from talking out of turn and may asked to leave the class should they fail to abide by these rules.

**Attendance Policy**

Absences must be excusable, resulting from a circumstance that is beyond the student’s control (e.g. illness, family crisis, necessary travel). You must provide us with a written statement (e-mail or note) regarding the reason for your absence. Otherwise, every unexcused will be deducted from the course grade (1 point per absence). Students missing classes should consult an instructor to inquire about missed assignments (i.e. in-class activities).

**Policy on Missed Exams and Deadlines:**

All unexcused late assignments will receive a 10% deduction per day late. Assignments turned in after answer keys are posted will be given no credit. We do not give make-ups for exams or assignments.

**Policy on Academic Dishonesty**

Copying the work of another student, sharing your work with others, plagiarism, and falsification of results will not be tolerated and can result in immediate disciplinary action, including the possibility of dismissal.

Students who violate these policies will receive an F for the course. Furthermore, students in violation of these policies will be sent before the Drexel Office of Judicial Affairs: <http://www.drexel.edu/judicial/default.html>. For Drexel’s policy on academic dishonesty, visit: http://www.drexel.edu/provost/policies/academic_dishonesty.asp.

**Students with Disabilities**

Students with disabilities requesting accommodations and services at Drexel University (e.g. extra time for exams), need to present a current accommodation verification letter (AVL) to the professor before accommodations can be made. This will need to be done 2 weeks in advance of the first exam (by Jan. 21).  AVL’s are issued by the Office of Disability Services (ODS).  For additional information, contact ODS at 3201 Arch St., Street, Suite 210, Philadelphia, PA  19104, ***215.895.1401*** (V), or ***215.895.2299*** (TTY). Or visit their website at [*www.drexel.edu/ods*](http://www.drexel.edu/edt/disability).

**ADD, DROP AND WITHDRAWAL POLICIES**

- You can **add** or **drop** this course until the end of week 1 (Sunday September 25 at 11:59 p.m.). For more on university policies relating to adding or dropping a course, see [http://drexel.edu/provost/policies/course-add-drop/](http://drexel.edu/provost/policies/course-add-drop/ ) . If you add this course after the start of the term, you are responsible for completing ALL work that you may have missed.
- The course **withdrawal** deadline is the Friday of week 7 (Friday November 11). You will have received some graded work prior to this deadline. If you have any questions about your progress at any time of the term, please contact me. If you choose to Withdraw, a “W” will be recorded in your transcript. For more on university policies relating to adding or dropping a course, see http://drexel.edu/provost/policies/course-withdrawal/%20/ .

**COURSE SCHEDULE**

| **Class session** | **Lecture topic** | **Assignments & Readings** |
| --- | --- | --- |
| 1 | Syllabus review^JR^  Intro to Bioinformatics lecture^GR^  The NCBI database—sequence retrieval, interacting with genomes, OMIM^JR^ | Begin in class assignment |
| 2 | -Genomes: mutation, drift, natural selection^JR^  -Computational methods^GR^ | -Homework #1 assigned |
| 3 | -Computational methods^GR^  -DNA sequence alignments^JR^  -DNA sequence alignment: Smith-Waterman algorithm (dynamic programming)^GR^ | -Homework #1 due |
| 4 | -DNA sequence alignment algorithms: Needleman-Wunsch algorithm (dynamic programming)^GR^  -BLAST^JR^  -Computational methods^GR^ | -Graduate student project proposals due |
| 5 | -Genome features^JR^  -Genome sequencing, assembly and annotation^JR^  -Computational methods^GR^ | -Homework #2 assigned |
| 6 | -Intro to Markov Chains and The Viterbi Algorithm (dynamic programming for genome annotation)^GR^  -The forward-backward Algorithm and the Baum-Welch Algorithm (dynamic programming) ^GR^ | -Homework #2 due |
| 7 | **-Midterm exam**  -Phylogenetics^JR^ | Midterm exam |
| 8 | -Phylogenetic algorithms^GR^  -Computational methods^GR^ | -Homework #3 assigned |
| 9 | -Molecular evolution^JR^  -Wrap up in class assignments | -Homework #3 due |
| 10 | -Graduate student project presentations | -Graduate student project final drafts due the last day of classes |

***FINAL EXAM: TBD***
