# Supplementary material for: Teaching Microbiome Analysis: From Design to Computation Through Inquiry
Source: Front Microbiol. 2020 Oct 29;11:528051. doi: 10.3389/fmicb.2020.528051 (PMC7658192; doi:10.3389/fmicb.2020.528051)
Supplement: Supplementary Syllabus 3 — Computational microbiome analysis syllabus (sometimes called Statistical Analysis of Genomics). [file Data_Sheet_3.doc]

**Statistical Analysis of Genomics**

**ECES 490/690**

3 credits

Mon 6 – 8:50 p.m.

Location: Bossone 605

**Instructor:**

Dr. Gail Rosen

gailr@ece.drexel.edu

215-895-0400

Bossone 403

Office hours: Tuesday 4-6

**Teaching Assistant:**

Stephen Woloszynek

sw424@drexel.edu

Bossone 325/TBA

Office hours: By Appointment

**Course Overview:**

This course will focus on developing the computational and database navigational skills required to analyze genomic data that have become available with the development of high throughput genomic technologies. Students will learn Python and R to analysis genomics. Many third party packages to analyze genomics such and assess the statistical significance of results will be learned. The goals will be achieved through lecture and lab exercises that focus on genomic databases, programming for importing and pre-processing genomic data, high performance programming for analysis of high-throughput metagenomic analyses, and use of high-performance computing for phylogenetic reconstruction.

**Statement of Expected Learning:**

1. An understanding of genomes, their features, how they vary, and the forces that drive this variation. This will be assessed through class tutorials and the class project.
2. To show competency in basic bioinformatics techniques related to genome annotation, sequence alignment, metagenomic analyses, and phylogenetics. The achievement of this objective will be assessed through in class tutorials and project.
3. To show a working knowledge of bioinformatics programming through unix scripting and Python and/or R. The achievement of this objective will be assessed through in class tutorials and class project.

**Course Reading Materials**

None required

Suggested: Python/Bash/R cookbooks and tutorials

**Expected letter grade-breakdown**

**A+** = 98% or more; **A** = 92 - 97.99%; **A-** = 90 - 91.99%

**B+** = 88-89.99%; **B** = 82 - 87.99%; **B-** = 80 - 81.99%

**C+** = 78-79.99%; **C** = 72 - 77.99%; **C-** = 70 - 71.99%

**D+** = 68-69.99%; **D** = 62 - 67.99%;

**F** = 59.99% or less

**Grading Policy:**

**ECES Undergraduates (ECES 490)**

***Lecture***

*20 pts* Weekly Quizzes and Quiz Questions

*30 pts* One Tutorial

*50 pts* Data Analysis Project

**ECES Graduates (ECES 690)**

***Lecture***

*20 pts* Weekly Quizzes and Quiz Questions

*30 pts* **Two** Tutorials

*50 pts* Data Analysis Project

**Details on specific assignments:**

Please see Project Guidelines Document

**General Code of Conduct:**

Students are expected to refrain from disruptive activity during class. Cell phones must be turned off or silenced (i.e. on vibrate). Text messaging and phone calls will are not allowed. Use of computers and electronic devices must be limited to note-taking or in-class computational exercises. Students must also refrain from talking out of turn and may asked to leave the class should they fail to abide by these rules.

**Attendance Policy**

Absences must be excusable, resulting from a circumstance that is beyond the student’s control (e.g. illness, family crisis, necessary travel). You must provide us with a written statement (e-mail or note) regarding the reason for your absence. Students missing classes should consult an instructor to inquire about missed assignments (i.e. in-class activities).

**Policy on Missed Exams and Deadlines:**

Generally speaking, we will not give make-up assignments or exams. As such, **excusable** absences or missed deadlines (see above) will require that we adjust the schedule, but you cannot skip assignments in this course.

All unexcused late assignments (turned in before the answer key is posted) will receive a 10% deduction per day late.

**Policy on Academic Dishonesty**

For Drexel’s policy on academic dishonesty, visit:

http://www.drexel.edu/provost/policies/academic_dishonesty.asp

Unless group/team activity is required, it is assumed that ALL work be solely that of the individual student whose name is associated with the work. ANY form of cheating (copying, plagiarizing, using another’s work, permitting another student to use your work, falsifying data, etc.) will not be tolerated and can result in immediate disciplinary action, including the possibility of dismissal.

Students who violate these policies (e.g. through cheating or plagiarism) may receive a 0 on the relevant assignment or, in more serious cases, may receive an F for the course. Furthermore, students in violation of these policies may be sent before the Drexel Office of Judicial Affairs:

<http://www.drexel.edu/judicial/default.html>

**Students with Disabilities**

Students with disabilities requesting accommodations and services at Drexel University (e.g. extra time for exams), need to present a current accommodation verification letter (AVL) to the professor before accommodations can be made. This will need to be done 2 weeks in advance of the first exam (by Jan. 21).  AVL’s are issued by the Office of Disability Services (ODS).  For additional information, contact ODS at 3201 Arch St., Street, Suite 210, Philadelphia, PA  19104, ***215.895.1401*** (V), or ***215.895.2299*** (TTY). Or visit their website at [*www.drexel.edu/ods*](http://www.drexel.edu/edt/disability).

**COURSE SCHEDULE**

| **Date** | **Lecture topic** | **Every week a tutorial assignment must be completed** |
| --- | --- | --- |
| January 10 | Syllabus review  Introduction to Metagenomics  Introduction to R  *Bioconductor, Remote BLAST, and Data structures in Phyloseq* | List of Tutorials given |
| January 17 | Introduction to Shell scripting and Biopython  *Shell Scripting and Python for accessing sequences via NCBI*  *Introduction to Proteus*  *Local Standalone BLAST* | **Choose Tutorial by January 19** |
| January 24 | Intro to Microbial Survey Analysis (aka 16S rRNA)  More Microbial Survey Analysis (using “meta-packages”)  *Introduction to QIIME*  *Vs. Dada2* | **Choose Project by January 26** |
| January 31 | “Meta-Packages” and their features  *MG-RAST*  *MEGAN* | Tutorials 1 and 2 |
| February 7 | Phylogenetics  *High-Performance Alignment and Tree Construction using CIPRES* | Tutorial 3 and 4 |
| February 14 | Introduction to Whole Genome Shotgun Analysis  Assembly and Binning  (MetaIDBA-UD, MetaBat) | Tutorial 5 |
| February 21 | Taxonomic Identification from WGS  *Choice of one “compositional read-by-read” and one abundance-estimation* | Tutorials 6 and 7 |
| February 28 | Functional annotation of microbial surveys  *Picrust*  *Tax4fun* | Tutorials 8 and 9 |
| March 7 | Functional annotation of metagenomes  *MUSiCC*  *DeSeq vs. EdgeR for differential abundance comparisons* | Tutorials 10 and 11 |
| March 14 | Functional annotation of metagenomes (protein families and metabolic pathways – overview)  *Project Presentations* | Tutorials 12 |

***Finals Week: More Project Presentations***
