# Supplementary material for: Teaching Microbiome Analysis: From Design to Computation Through Inquiry
Source: Front Microbiol. 2020 Oct 29;11:528051. doi: 10.3389/fmicb.2020.528051 (PMC7658192; doi:10.3389/fmicb.2020.528051)
Supplement: Supplementary Questions — The questions that were a part of the IRB process at the beginning of the project. Some questions that assessed outdated topics were omitted. Also, questions that did not have a statistically significant difference between groups were omitted. [file Data_Sheet_4.docx]

# Supplementary Material

The following are the summative assessment pre/post-questions decided at the beginning of the project. Due to changes in course development, not all questions were given to students or not enough answers from students were collected resulting in high p-values. A breakdown of omission vs. p-values for all the questions are shown:

1. What is the difference between UPGMA and Neighbor Joining (NJ) and what assumption does UPGMA make about the molecular clock? – **Was omitted**
2. What is a Standard Flowgram File and what type of DNA sequencer outputs it? – **p-value=0.019**
3. How would you convert a SFF file to a FASTA file? – **p-value=0.021**
4. What is the difference betweeen PCA (Principal components analysis) and PCoA (Principal coordinates analysis)? – **p-value=0.030**
5. What are the trade-offs of supervised learning algorithms (trade-off of random forests vs. support vector machines vs. bayes classifiers)? – **p-value=0.001**
6. Genome sizes for a given species or taxon vary, often considerably. Describe why metatranscriptomic reads need to be normalized, especially for downstream analysis. – **Was omitted**
7. What do techniques like PCoA (principal coordinates analysis) and t-SNE do? – **Was omitted**
8. What is the difference between taxonomic classification vs. binning? Please state which one uses supervised techniques and which one unsupervised techniques. – **Was omitted**
9. To learn particular features relevant to a classification, what type of machine learning methods should be used? – **p-value=0.054**
10. Please describe the general process to isolate DNA of a sample? – **p-value=0.648**
11. Please describe the general PCR (polymerase chain reaction) process? – **Was omitted**
12. When should PCR be used? – **Was omitted**
13. What is the 16S rRNA gene and why is it important? – **p-value=0.441**
14. Name at least two ways that you can annotate WGS (whole-genome shot sequencing_ reads with functional annotation? – **p-value=0.004**
15. Describe the difference between phylogenetic tree reconstruction methods? – **p-value=0.001**
16. Why is metagenomic assembly of isolate genomes difficult? – **Was omitted**
17. What does sequence alignment try to accomplish. – **Was omitted**
18. Which method can a) capture the potential of functions present in the environment and b) which method captures the actual express of functions being expressed? (match "metagenomics" and "metatranscriptomics" with these statements) – **Was omitted**
19. What does Unifrac accomplish? – **p-value=0.168**
20. How is the Unifrac distance metric different from other distance metrics? – **Was omitted**
