# Supplementary material for: Teaching Microbiome Analysis: From Design to Computation Through Inquiry
Source: Front Microbiol. 2020 Oct 29;11:528051. doi: 10.3389/fmicb.2020.528051 (PMC7658192; doi:10.3389/fmicb.2020.528051)
Supplement: Supplementary Evaluator Report — A qualitative report of formative assessments of the classes. [file Data_Sheet_5.pdf]

Evaluator Qualitative Report

Perceptions and Understanding

In order to examine learners' perceptions and understanding of the content learned across the series of the three courses, a survey (formative assessment) was administered in weeks 3 and 6. Each survey asked the same five questions. The information collected from the survey was used to inform the course activities on an ongoing basis.

Thematic analysis of the student responses from the surveys was conducted using MAXQDA software. Two researchers independently coded the qualitative data to identify boarder themes and categories. The results were then discussed until 100% agreement was reached between the raters. Table 4 and Table 3 below present a snapshot of student responses to the questions in weeks 3 and 6 for both Fall 2016 and Winter 2017 quarters.

Table 4
*Student Perceptions and Understanding for Fall 2016 Bioinformatics: N=18 (*8 students from an electrical/computer engineering background, 7 students from a Biosciences background, and 3 Biomedical Engineers (hybrid background)).

Question	Themes for Week 3	Example Responses	Themes for Week 6	Example Responses
**…which activities were more useful and why?**	Homework; algorithm; programming; databases	*"NCBI databases; alignment scoring by hand; a lot of valuable information"*<br><br>*"the sequence alignment tracebook or the board; visualizing the code"*	Hidden Markov Models; sequences; Homework; in-class practice; algorithms.	*"...learning about Hidden Markov Models"*<br><br>*"Programming: exposure to languages…"*<br><br>*"How to pull genetic sequences out of NCBI".*<br><br>*"Whiteboard activities, examples, homework assignments".*

**…which activities were least useful and why?**	Lectures; programming assignment; coding; biology concepts.	*"Python homework assignment…"*<br><br>*"understanding code"*<br><br>*"R coding because I have very little background coding."*	Homework; coding; lectures; algorithms; bootstrapping; nothing; Biology information	*"Using R, bootstrapping by hand was confusing…"*<br><br>*"Biology background because I was familiar with this."*<br><br>*"Algorithm lectures implementing it myself would help much more…"*
**…what did you learn the most about and why?**	NCBI database; sequence alignment; algorithm; phylogenetics	*"Learned the most about NSBA databases, their uses and how we can optimize the information."*<br><br>*"DNA, RNA structures and their characteristics, Bio vocabulary and the computational algorithm used in Bioinformatics."*<br><br>*"How to search NCBI databases with coding programs."*	NCBI database; coding; Markov models; HMMS; biology content.	*"Providing phylogenetic trees because it was covered in depth."*<br><br>*"NCBI was useful tool."*<br><br>*"Basics of genetics and phylogenetics"*<br><br>*"Hidden Markov"*
**…what did you have the most trouble learning and shy?**	Fast pace; programming language; lack of background; NCBI; sequence alignment.	*"Python and R; limited programming knowledge, fast-paced lectures."*<br><br>*" sequence alignment and problems were explained too quickly."*<br><br>*"Writing Python code for searching NCBI"*	coding; Bootstrapping	*"Using R, bootstrapping by hand was confusing."*<br><br>*"algorithms for analyzing HMMs"*<br><br>*"coding because of lack of background"*<br><br>*"Python, R any coding language"*
…**what would you like to see changed?**	More explanation and exercise.	*"more explanations and exercise for how functions work."*<br><br>*"More assignments to judge our performance."*	More practice; more explanation.	*"Give the course more than once a week, spend more time explaining fewer topics, give homework assignments."*

		"More prewritten code to follow."		*"More coders explained"*<br><br>*"More frequent shorter homework"*<br><br>*"more interactive example of the HMM"*

Table 5
Student Perceptions and Understanding for Winter Computational Microbiome Analysis 2017
N=8 (4 electrical/computer engineers, 2 Bioscience students, and 2 Biomedical Engineers (hybrid background))

Question	Themes for Week 3	Example Responses	Themes for Week 6	Example Responses
**…which activities were more useful and why?**	Tutorials; programming languages; lectures and demonstrations.	*"…lectures because they are informative easy to follow."*<br><br>*"…coding demonstrations and R tutorials."*<br><br>*" Using Bio-Python and R."*	Tutorials; learning how to use tools.	*"The tutorials have been useful to learn about the different packages."*<br><br>*"Doing the tutorials along with the students presenting them."*
**…which activities were least useful and why?**	Faced-paced tutorials and exercises.	*"Tutorials for R were really fast-paced."*<br><br>*"Coding exercises sometimes go too quick."*	Quizzes	*"The quizzes because there is a lot of information each session and it would take time away from the lesson on the tutorial."*<br><br>*"The quizzes were least useful because the answers were not reviewed in weeks 3-5."*
**…what did you learn the most about and why?**	R; metagenomics; biology concepts.	*"I have learned the most about R because my knowledge was very limited prior to this."*<br><br>*"..microbiology and ecology."*<br><br>*"Metagenomics! This is my first bioinformatics course so learning what this*	Metagenomics; tools (Proteus, MEGAN).	*"I have learned the most about how to use Proteus because it is needed for the project and tutorial."*<br><br>*"I learned the most about the varieties of tools available for metagenomics analysis and the nuances in algorithms."*

		data do and how we analyze it was new to me.		
**…what did you have the most trouble learning and shy?**	Coding; biology	*"The programing. I am a biology major, so I had background in that, but I am not as comfortable with programming."*<br><br>*"Everything biology related. I don't have background in it."*	Biology concepts (lack of background).	*"I had the most trouble with understanding species diversity measures and how species level classification can be compared between different tools."*<br><br>*"…anything bio related. I'm an engineering student with no bio classes."*
**…what would you like to see changed?**	More explanation; More practice exercises.	*"Move basic instruction for biology related material"*<br><br>*"More explanation of the purposes of the packages on R."*	Longer class duration; more programming.	"*It would be helpful to have more general programming/how to use Proteus in general …tutorials."*

As can be seen from Tables 4 and 5, in both quarters students reported having had different experiences while engaging in the course activities and materials. As expected, students with little or no biology background had the most trouble understanding biology-related concepts. Similarly, students with little or no engineering background had the most trouble learning different programing or coding languages. Overall, students found the courses useful, indicating that they learned much about different programming languages (eg. R, Python), databases (eg. NCBI), algorithms, and sequence alignments. For both quarters, in response to the changes they would like to see in the course, the participants indicated that they would like to have more practice exercises and more detailed explanation of the course content.

Post-Questionnaire (Summative Evaluation)

At the end of each quarter, perception and experience test was administered in addition to quality and impact questions. There were three questions asking students` expectations in relation to their own expectations and other science/engineering courses. There were also seven questions asked about the impact of the current course on Genetics, Ecology, Bioinformatics, Metagenomics, Hypothesis development, Experimental Design, and manipulating data. Open-ended responses were coded using MaxQDA software by two different coders separately. Then, the codes were discussed to reach out 100% agreements on common themes.

In the following sections, themes and examples will be discussed.

Perceptions and Experiences

Table 6 below reviews the students` perceptions and experiences toward Bioinformatics and Molecular Ecology Lab.

Table 6

Students' perceptions and experiences toward Bioinformatics and Molecular Ecology Lab.

Questions	Themes	Example Responses
**1. … how well did the course meet your expectations..?**	a) Met the expectations very well<br><br>b) Somewhat met my expectations	*a) "…the course exceeded my expectations",*<br><br>*"The course was really helpful and met all of my expectations"*<br><br>*b) "I expected the course to feature more biology but have been surprised to find it heavily favored coding",*<br><br>*"I expected to learn some practical skills that I can use such as a script to sequence alignments but*

		this course taught a lot about background theory of these algorithms."
**2. Describe the activities that made the most impact on your experience in the course…?**	Coding activities, BLAST searches, Hidden Markov Models, homework assignments, NCBI database	*"…coding activities most difficult to understand but most rewarding,"*<br>*"BLAST was really helpful"*<br>*"…The Markov and the sequence matrix were good"*<br>*"…use of NCBI was great"*
**3. How was this course different than other science/engineering courses that you have taken…?**	a) More hybrid than other courses<br>b) More programming	*a) "The fusion of disciplines is ready apparent", "This course is more hybrid than all other engineering science courses I`m taken. Requiring understanding of two fields to apply them in bioinformatics*<br>*b) "…there was also more programming then I was used to", "It was a programming course"*

As to table 6 question 1, many of the students described the course as a challenging but rewarding course that met their expectations. Some students also mentioned that the course somewhat met their expectations. These group of students had either background in programming or biology. The students with backgrounds in biology found programming part challenging and the students with programming background found biology challenging. For

questions 2 and 3 the most frequent themes are presented, and example responses are given in the very right column. Table 7 below reviews the students` perceptions and experiences toward the Computational Microbiome Analysis course.

Table 7

The students' perceptions and experiences toward Computational Microbiome Analysis course.

Questions	Themes	Example Responses
1. **… how well did the course meet your expectations..?**	Met highly my expectations	*"It definitely met my expectations because I gained knowledge in bioinformatics tools"*<br>*"The course exceeded my expectations for the topics"*
2. **Describe the activities that made the most impact on your experience in the course…?**	a) R coding language<br>b) tutorials	*a) "I found the R exercises from the first few weeks really helpful…"*<br>*"R material…"*<br>*b) "Tutorials – had to become acquaintance with program tools."*<br>*"The tutorial were very helpful…"*
3. **How was this course different than other science/engineering courses that you have taken…?**	a) More project-based<br>b) Covered more Biology	*a) "…It is more project-based than most biology classes"*<br>*"It was solely project-based"*<br>*b) "Bio was a bit of a wakeup call and made*

		the class different than any other course"

As for the students' perceptions toward the Computational Microbiome Analysis course, a great majority of them found the course very useful that highly met their expectations. The related examples are in the very right column. As for the activities coding activities and especially R coding activities were desirable. Some students also mentioned tutorials that one example is also given in Table 7. The students also found this course different from the other science/ engineering courses in the sense that it was more project based or practical. Some students also talked about biology background which builds enough background for coding.

Quality and Impact Questions

At the end of each course quality and impact questions asked about the impact of the current course on Genetics, Ecology, Bioinformatics, Metagenomics, Hypothesis development, Experimental Design, and manipulating data. Tables 8 and 9 reviews the frequent answers that students had given to impact questions. Some examples are also given for clarifying the themes.

Table 8

Students' perceptions toward quality and impact of Bioinformatics and Molecular Ecology Lab

Questions	Themes	Example Responses
**1. …impact your knowledge of Genetics?**	Increased knowledge of Genetics	*"It increased my knowledge of genetics…"*<br><br>*"This case allowed me to learn more about using genetic databases"*<br><br>*"It has increased it a I can now construct phylogenetic trees"*

**2. …impact your knowledge of Ecology?**	Poorly/very little	*"Not a great extent, most focus was made on biology"*<br><br>*"This course only added a small amount to my knowledge of ecology…"*<br><br>*"Didn't learn much about ecology…"*
**3. …impact your knowledge of Bioinformatics?**	Significantly increased	*"It significantly increased my knowledge of bioinformatics …"*<br><br>*"This course greatly expanded my knowledge and general exposure to bioinformatics…"*
**4. …impact your knowledge of Metagenomics?**	a) A small amount/poorly<br><br>b) Medium	*a) "Decent extent"*<br><br>*"This did not impact any knowledge about genetics"*<br><br>*"A small amount/poorly"*<br><br>*b) This course mentions metagenomics but does not go in depth about how it relates to bioinformatics"*
**5. …impact your knowledge of**	Not at all	*"My knowledge of hypothesis development stayed the same"*<br><br>*"Not a great extent"*

**Hypothesis Development?**		*"1 means not at all. No hypothesis development was practiced"*
**6. …impact your knowledge of Experimental Design?**	Not at all	*"Did not design experiments"*<br><br>*"none"*<br><br>*"not at all"*
**7. …impact your knowledge manipulating data?**	A large amount	*"My knowledge of manipulating data increased significantly with programs such as SeaView"*<br><br>*"This course greatly impacted my knowledge by manipulating data…"*

As can be seen in Table 8, the last questions ask about the impact of the course on manipulating data. Many students believed that the course impacted their knowledge of manipulating data significantly and they gave many interesting examples which are mentioned above in the example response column. Some other interesting examples are about coding, bootstrapping and applying class knowledge to outside. For example, one student wrote:

> " This course greatly impacted my knowledge by manipulating data because I was able to use my own data to put topics learned in class to use outside the class"

Table 9

Students` perceptions toward quality and impact of Metagenomics

Questions	Themes	Example Responses

**1. ….impact your knowledge of Genetics?**	Significantly increased	*"It heightened my knowledge of the field"*<br><br>*"A lot! I learned a lot of bio behind it."*
**1. …impact your knowledge of Ecology?**	Not at all/ poorly	*"Not really, ecology was barely brought up"*<br>*"not too much ecology specific"*<br><br>*"Decent. I learned how certain bacteria can be mapped based on the sample locations."*
**2. …impact your knowledge of Bioinformatics?**	A lot/ significantly	*"A lot! I was able to learn many bioinformatics tools."*<br><br>*"it heightened it considering I have of the most common software used."*
**3. …impact your knowledge of Metagenomics?**	A lot/ significantly	*"Significantly, we went through a metagenomics pipeline for our project."*<br><br>*"Greatly, topics were mostly specific to metagenomics."*
**4. …impact your knowledge of Hypothesis Development?**	a) Somewhat<br><br><br><br>b) A little/poorly	*a) "Somewhat, hypothesis development was expected in the project."*<br><br>*"I did not hypothesize much data but I am fairly comfortable with it as is."*<br><br>*b) "I learned about it a bit with Steve's lecture, I think"*<br><br>*"Not too great."*

**5. …impact your knowledge of Experimental Design?**	a) Somewhat<br><br><br><br><br><br><br><br><br>b) Not at all/poorly	*a) "Somewhat with the project."*<br><br>*"Not a ton but in the project I came to understand some of what is important with SRA documentation, etc."*<br><br>*b) "not too great"*<br><br>*"none"*
**6. …impact your knowledge manipulating data?**	A lot/ significantly	*"Heightened my understanding because of the normalization and conversion methods."*<br><br>*"I learned about general manipulation with our project"*

Generally, the students believe that Bioinformatics and Molecular Ecology Lab had a great impact on their knowledge of Genetics, Bioinformatics, and Manipulating data. However, they did not gain such a great knowledge on Ecology, Metagenomics, Hypothesis Development and Experimental Design.

As to the Computationa Microbiome Analysis course, the students also believed that the course increased their knowledge of Genetics, Bioinformatics, Metagenomics, and Manipulating Data significantly. Somehow similar to Bioinformatics and Molecular Ecology Lab, the Computational Microbiome Analysis course did not increase their knowledge of ecology, Metagenomics, Hypothesis Development, and Experimental Design. The only difference of first two courses with the third one is in the impact of Metagenomics that Bioinformatics and

Molecular Ecology Lab failed to address Metagenomics while the Computational Microbiome Analysis course significantly increased the knowledge of Metagenomics among the students.
